# Development of functional hydroxyethyl cellulose-based composite films for food packaging applications

**DOI:** 10.3389/fbioe.2022.989893

**Published:** 2022-09-29

**Authors:** Xueqin Zhang, Haoqi Guo, Wenhan Luo, Guojian Chen, Naiyu Xiao, Gengsheng Xiao, Chuanfu Liu

**Affiliations:** ^1^ College of Light Industry and Food Technology, Zhongkai University of Agriculture and Engineering, Guangzhou, China; ^2^ Academy of Contemporary Agricultural Engineering Innovations, Zhongkai University of Agriculture and Engineering, Guangzhou, China; ^3^ Guangdong Key Laboratory of Science and Technology of Lingnan Specialty Food, Zhongkai University of Agriculture and Engineering, Guangzhou, China; ^4^ State Key Laboratory of Pulp and Paper Engineering, South China University of Technology, Guangzhou, China

**Keywords:** hydroxyethyl cellulose, chemical cross-linking, composite film, UV-shielding, antibacterial activity

## Abstract

Cellulose-based functional composite films can be a good substitute for conventional plastic packaging to ensure food safety. In this study, the semi-transparent, mechanical strengthened, UV-shielding, antibacterial and biocompatible films were developed from hydroxyethyl cellulose Polyvinyl alcohol (PVA) and ε-polylysine (ε-PL) were respectively used as reinforcing agent and antibacterial agent, and chemical cross-linking among these three components were constructed using epichlorohydrin The maximum tensile strength and elongation at break were 95.9 ± 4.1 MPa and 148.8 ± 2.6%, respectively. TG-FTIR and XRD analyses indicated that chemical structure of the composite films could be well controlled by varying component proportion. From UV-Vis analysis, the optimum values of the percentage of blocking from UV-A and UV-B and ultraviolet protection factor values were 98.35%, 99.99% and 60.25, respectively. Additionally, the composite films exhibited good water vapor permeability, swelling behavior, antibacterial activity and biocompatibility. In terms of these properties, the shelf life of grapes could be extended to 6 days after packing with the composite film.

## Graphical Abstract

**Figure:**
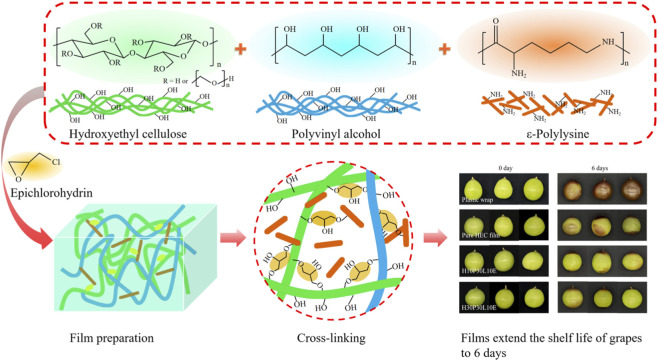

## 1 Introduction

In the past decades, food spoilage caused by microorganism contamination has been one of the most serious global crises, which greatly threatens the health of human beings (Xu J. H. et al., 2020; Xie et al., 2020). Traditionally, plastic films based polymers are widely applied for food packaging due to their favorable physiochemical properties and low cost (Hu et al., 2016; Khosravi et al., 2020; Sirviö et al., 2020). However, plastic films are non-degradable, which lead to a number of environmental problems. More, the inactivity of plastic films requires additional chemical preservative to inhibit the growth of microorganism and prolong the shelf life of food, especially for food with high moisture content and proteins (Kritchenkov et al., 2019). Additionally, plastic films have low permeability, resulting in dewing phenomenon of fresh fruits and vegetables at low temperature (Zhu et al., 2021). Recently, environmentally friendly natural and sustainable biopolymers give rise to more and more attentions and are promising to replace traditional polymeric plastics (Kritchenkov et al., 2019; Mehta and Kumar 2019). As we know that the composite technique is the most feasible method to incorporate the advantageous performances of each component and simultaneously extend the applications of the obtained composite materials (Hu et al., 2018; Kurek et al., 2018). Therefore, it is promising to fabricate functional biopolymer composites with good antibacterial ability and permeability.

Cellulose, a natural biopolymer, is widely used in food packaging area (Shahmohammadi Jebel and Almasi 2016; Noshirvani et al., 2017). Cellulose and its derivatives, like bacterial cellulose (Atta et al., 2022a; Manan et al., 2022), hydroxyethyl cellulose (HEC) (Jo et al., 2021), carboxymethyl cellulose (Atta et al., 2022b) etc., are explored for their potential applications in food packaging area to improve the quality of food. HEC, a common derivative of cellulose, is a water-soluble polysaccharide that exhibits good film-forming ability, pseudoplastic solution behavior, tolerates salts, and retains water (Şen and Kahraman 2018; Lu et al., 2020). HEC is synthesized by nucleophilic ring opening of ethylene oxide, by the hydroxyl anions on the anhydroglucose ring of cellulose (Şen and Kahraman 2018). Due to its good physicochemical properties, HEC is widely used in industrial applications such as in food products, adhesives, paints, textiles and paper (Joubert et al., 2015; Zhao et al., 2016; Lu et al., 2020). However, as compared with other cellulose ether derivatives with good film-forming ability, HEC is mainly used as additives in combination with other polymers (Jo et al., 2021; Zhang et al., 2022). As such the physical behavior of HEC films has not been fully characterized, it would be interest and necessary to enhance current knowledge of the physical behavior of HEC films for packaging use.

Cross-linking cellulose with other polymers provides an effective way to obtain films with desired properties. Among these polymers, polyvinyl alcohol (PVA) is brought into focus due to its outstanding optical transparency, film-forming ability and oxygen barrier ability, broadening its application in textile, cosmetics, pharmaceuticals, and food packaging areas (Wahid et al., 2019). For example, Sirviö et al. (Sirviö et al., 2015) prepared acetal-bonding cross-linked PVA films with 2-fold tensile strength; Takeno et al. (Takeno et al., 2020) synthesized cellulose nanofiber/PVA hydrogel films cross-linked by a freezing/thawing method and borax, and the dual cross-linking resulted in remarkable increase of mechanical properties; Song et al. (Song et al., 2018) developed chemical cross-linked PVA/cellulose nanocrystal composite films with high structural stability by spraying Fenton reagent as an initiator. In terms of these, biopolymer cross-linked with PVA provides an efficient way to prepare composite films with promising properties. It would be of great interest to develop HEC/PVA films by cross-linking method.

Packaging with antibacterial ability is classified as a subset of active packaging that extends the shelf life of food with special add-ons in packing or in-pack films. Recently, the incorporation of natural bioactive compounds into bio-based composite films has achieved great focus (Wei et al., 2018; Wu et al., 2019; Li et al., 2020). By using natural agents, the addition amount of synthetic chemical additives can be greatly decreased to reduce the potential health risks. ε-Polylysine (ε-PL), a natural cationic polypeptide composed of 25–35 homogeneous l-lysine, is a biodegradable and water-soluble bacteriostatic agent with broad antimicrobial activity (Gao et al., 2014; Xu J. H. et al., 2020; Li et al., 2020). It has been proved for use in cooked or sushi rice by Food and Drug Administration. It has found that the addition of ε-PL to polymer matrix can develop the antibacterial ability of chitosan, proteins or alginate (Wu et al., 2019; Xu J. H. et al., 2020). To the best of our knowledge, it has few reports to date about ε-PL in combination with HEC and PVA.

Considering the good film-forming properties of HEC and PVA, and the antibacterial activity of ε-PL, the aim of this study was to develop a novel ternary HEC/PVA/ε-PL composite film with improved mechanical properties, UV-shielding, antibacterial activity and biocompatibility by chemical cross-linking with epichlorohydrin (ECH). The physicochemical, morphological, and biological properties of the composite films were thoroughly characterized. Also the composite films were applied as the promising packaging material to extend the shelf life of green grapes.

## 2 Materials and methods

### 2.1 Materials

HEC (1500–2500 mPa s), PVA, ECH and ε-PL were purchased from Shanghai Macklin Biochemical Co., Ltd. (Shanghai, China). All other reagents were of analytical grade and were commercially available.

### 2.2 Preparation of the chemical cross-linked composite films

Firstly, 1 g HEC was dissolved in 0.25 M NaOH (25 ml) at room temperature to obtain solution. Then a specific amount of PVA was dissolved in 25 ml hot water (Supplementary Table S1), and NaOH was added to obtain PVA solution with NaOH concentration of 0.25 M. After that, the above two solutions were mixed and cross-linked by ECH at 50°C for 6 h. Then ε-PL was added, followed by stirring at 50°C for another 1 h. The homogenous mixture was poured into dishes and dried at room temperature. The effects of the contents of PVA, ECH and ε-PL on the properties of the composite films were investigated. The composite films were designated as HxPyLzE, where H, P, L and E denoted the components of HEC, PVA, ε-PL and ECH, respectively, and x, y and z denoted the contents of PVA, ε-PL and ECH, respectively.

### 2.3 Characterization

Fourier-transform infrared (FT-IR) spectroscopy was conducted on Vertex 70 spectrometer (Bruker, Germany) at a resolution of 4 cm^−1^ with 32 scans per minute within the wavenumber range of 4000–400 cm^−1^.

Thermogravimetric-FTIR (TG-FTIR) measurement was carried out with a Bruker TGA-IR/NEZTSCH STA 449 F5 analyzer. Samples (∼10 mg) were placed in an aluminum crucible, and heated from the room temperature to 600°C in a 50 ml/min flow of nitrogen.

X-ray diffraction (XRD) pattern was characterized on a D/max-III X-ray diffractometer (Japan) equipped with nickel filtered Cu Kα radiation. The diffraction angle (2θ) was 5–80°.

The UV-Vis spectra and the transmittance of the composite films were recorded on a TU-1810 spectrophotometer (Beijing, China) in the range of 200–900 nm. Films were cut into strips and pasted on the surface of quartz pool. The ultraviolet protection factor (UPF) was applied to evaluate how much the material decrease UV exposure, calculated as follows (Wang et al., 2017):

$$UV\;protection\;factor\;\left(UPF\right)=\frac{\int_{280}^{400}E\left(\lambda \right)S\left(\lambda \right)d\lambda }{\int_{280}^{400}E\left(\lambda \right)S\left(\lambda \right)T\left(\lambda \right)d\lambda }$$ (1)

where E(λ) is the relative erythema action spectrum, S(λ) is the spectral irradiance (Wm^−2^ nm^−1^), T(λ) is average spectral transmittance of film, dλ is bandwidth, and λ is wavelength.

The percentage blocking for UV-A (320–400 nm) was calculated as follows:

$$UV-A\;blocking\;\left(\%\right)=100-\frac{\int_{320}^{400}T\left(\lambda \right)d\lambda }{\int_{320}^{400}d\lambda }\left(\%\right)$$ (2)

The percentage blocking for UV-B (280–320 nm) was calculated as follows:

$$UV-B\;blocking\;\left(\%\right)=100-\frac{\int_{280}^{320}T\left(\lambda \right)d\lambda }{\int_{280}^{320}d\lambda }\left(\%\right)$$ (3)

Scanning electron microscopy (SEM, Zeiss Sigma 500) with an accelerating voltage of 10 kV was used to characterize the cross-section morphology of the composite films. Before characterization, films were sputter-coated with a thin layer gold.

Film thickness was performed on a micrometer (Lorentzen and Wettre) with precision of 0.01 m. Each film was measured at seven different locations to obtain the mean value.

Mechanical properties were measured by an Instron Universal Testing Machine 5566 based on the “ASTM D882-12” standard method. Films were cut to 10 mm × 70 mm rectangular strips and tested with five times for each sample. The grips length was 30 mm, and the stretching rate was 4 mm/min.

Water vapor permeability (WVP) was measured gravimetrically according to Kurek et al. (Kurek et al., 2018). Films were sealed on a test vessel (diameter of 2.8 cm) containing 40 g dried silica gel, and then weighted. After that, the test vessel was placed in a desiccator containing distilled water at 20°C. The bottles were weighted periodically at intervals of 24 h for 7 days. The WVP value was calculated as follows:

$$WVP=\frac{w\times L}{t\times A\times ∆p}$$ (4)

where w is the weight gained (g), L is the film thickness (m), t is the elapsed time (s), A is the film permeation area (m^2^), and ΔP is 2339 Pa at 20°C.

Gravimetric method was applied to measure swelling properties. Films were cut into 30 mm × 5 mm specimens and dried until constant weight. The specimens were soaked in deionized water for 24 h, and then removed the water on the surface using filter paper. The water swelling ratio was calculated as follows:

$$Swelling\;ratio=\frac{M_{2}-M_{1}}{M_{1}}\times 100\%$$ (5)

where *M*_1_ and *M*_2_ are the masses of the films before and after soaking for 24 h. Each film was measured three times to obtain a reasonable average.

The antibacterial activities of the composite films against *E. coli* and *S. aureus* was determined by agar diffusion method (Fu et al., 2015). Briefly, films were cut into 15 mm diameter disks, and placed on *E. coli* and *S. aureus* cultured agar plates, and then were incubated at 37°C for 24 h. The appearance of a clear area below or around the film was considered to be positive for the antibacterial activity, and the bacterial inhibition zone (*W*_inh_) was calculated as follows:

$$W_{inh}=\frac{d_{1}-d_{2}}{2}$$ (6)

where *d*_1_ is the total diameter of the inhibition zone and the film, and *d*_2_ is the diameter of the film (15 mm).

*In vitro* cytotoxicity of the synthesized composite films was determined according to ISO10993 method. Firstly, film was cut into disk with diameter of 8 mm, and sterilized using ultraviolet radiation for 0.5 h and immersed into DMEM for 24 h. Then 200 μL of 3T3 mouse fibroblasts in DMEM (1.25 × 10^5^ cells/mL) was added in each 96-well plate and incubated at 37°C and 5% CO_2_ for 24 h. After that film was placed into DMEM with cells to cultivate for 24 and 48 h. After incubation, 200 μl of DMSO was added and shaken at room temperature. The optical density was measured at 570 nm with a microplate reader. All experiments were performed in triplicate. The absorbance values were expressed as a percentage of the control, representing 100% cell viability and Wells without composite film served as control (untreated).

The application of the prepared composite films in food preservation was studied using green grapes purchased from local fruit store. Uniform green grapes were singly packed and sealed in plastic wrap, pure HEC film, H10P30L10E and H30P30L10E, and three grapes were used for each film. All the samples were stored at 25°C and 55% RH. All experiments were performed in triplicate.

Statistical analysis was performed using SPSS statistical software 20.0 by analysis of variance (ANOVA). Data were expressed as mean ± standard deviation. The level of *p* ≤ 0.05 is used to evaluate the significant differences between two samples.

## 3 Results and discussion

### 3.1 Optimization and mechanical properties of the composite films

**SCHEME 1:**
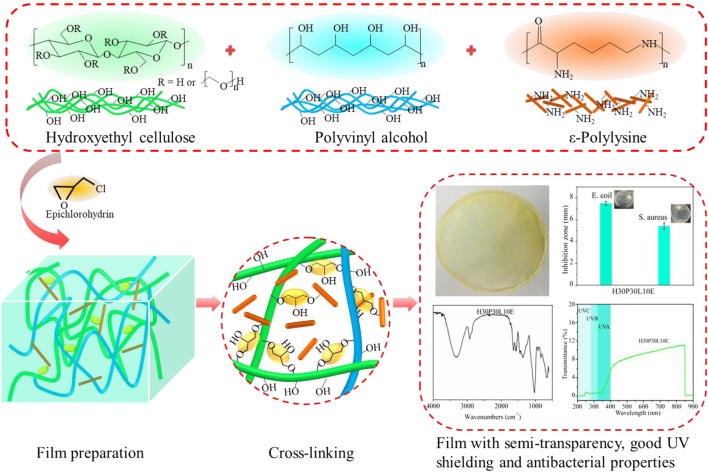
Process for the preparation of HEC-based composite films.

**FIGURE 1:**
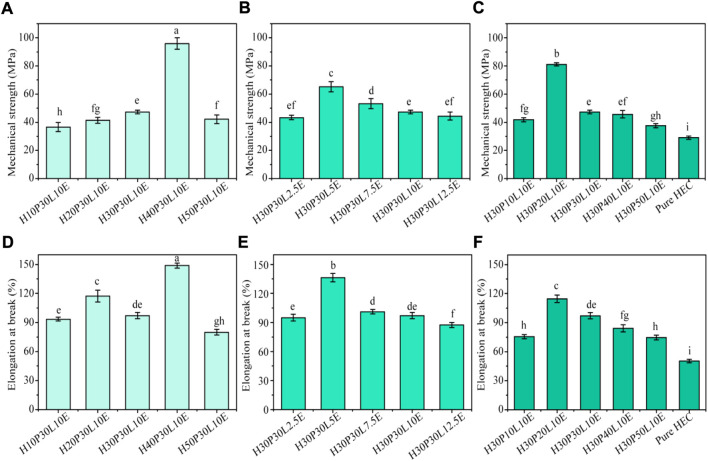
Tensile strength and elongation at break of the composite films with different contents of PVA **(A,D)**, ECH **(B,E)** and ε-PL **(C,F)**. All values in the same figure with different superscripts are significantly different (*p* ≤ 0.05).

Mechanical properties play an important role in packaging area. In this study, HEC was firstly reinforced by PVA and then cross-linked with ECH to prepare films with good mechanical performances (Scheme 1), and the results are shown in Figure 1. From Figure 1A, increasing PVA content from 10% to 40%, the mechanical strength of the composite films increased from 36.6 ± 3.3 MPa to 95.9 ± 4.1 MPa. This could be attributed to the increased cross-linking reaction between HEC and PVA. When further increase PVA content to 50%, the mechanical strength of the composite films dramatically decreased, probably due to the inherent low mechanical strength of PVA. From Figure 1D, increasing PVA content from 10% to 50%, the elongation at break of the composite films obtained an optimum value (148.8 ± 2.6%) with 40% PVA, and then decreased. This result indicated that the over-plasticization of PVA is not conducive to the improvement of the flexibility of film. From Figures 1B,E, the mechanical strength and elongation at break of the composite films presented same variation tendency by controlling ECH content. Specifically, increasing ECH content from 2.5% to 5%, the mechanical strength and elongation at break of the composite films increased from 43.4 ± 1.7 MPa and 94.9.8 ± 3.6% to 65.3 ± 3.5 MPa and 136.3 ± 4.4%, respectively. When further increase ECH content to 12.5%, the mechanical strength and elongation at break of the composite films decreased to 44.4 ± 2.8 MPa and 87.5 ± 2.5%, respectively. According to previous studies (Zhao et al., 2016; Kurek et al., 2018), there were three putative cross-linking reactions in this case: the–OH groups from HEC molecules could be cross-linked with the–OH groups from PVA molecules to form cross-linked structure, the–OH groups from HEC molecules could form inter-molecular cross-linked structure with the other HEC molecules, and the–OH groups from PVA molecules could form inter-molecule cross-linked structure with the other PVA molecules. In terms of these, the cross-linking reaction for the composite films is complicated because of the three potential competitive reactions.

Therefore, in this study, excessive cross-linking agent is not good to improve the mechanical properties of the composite films, which could be ascribed to the competitive side-cross-linking reactions. Similarly, this conclusion can also be used to explain that excessive ε-PL is not good for the development of the mechanical properties of the composite films (Figure 1C and Figure 1F).

### 3.2 FTIR, thermal ability and TG-FTIR analyses of the composite films

**FIGURE 2:**
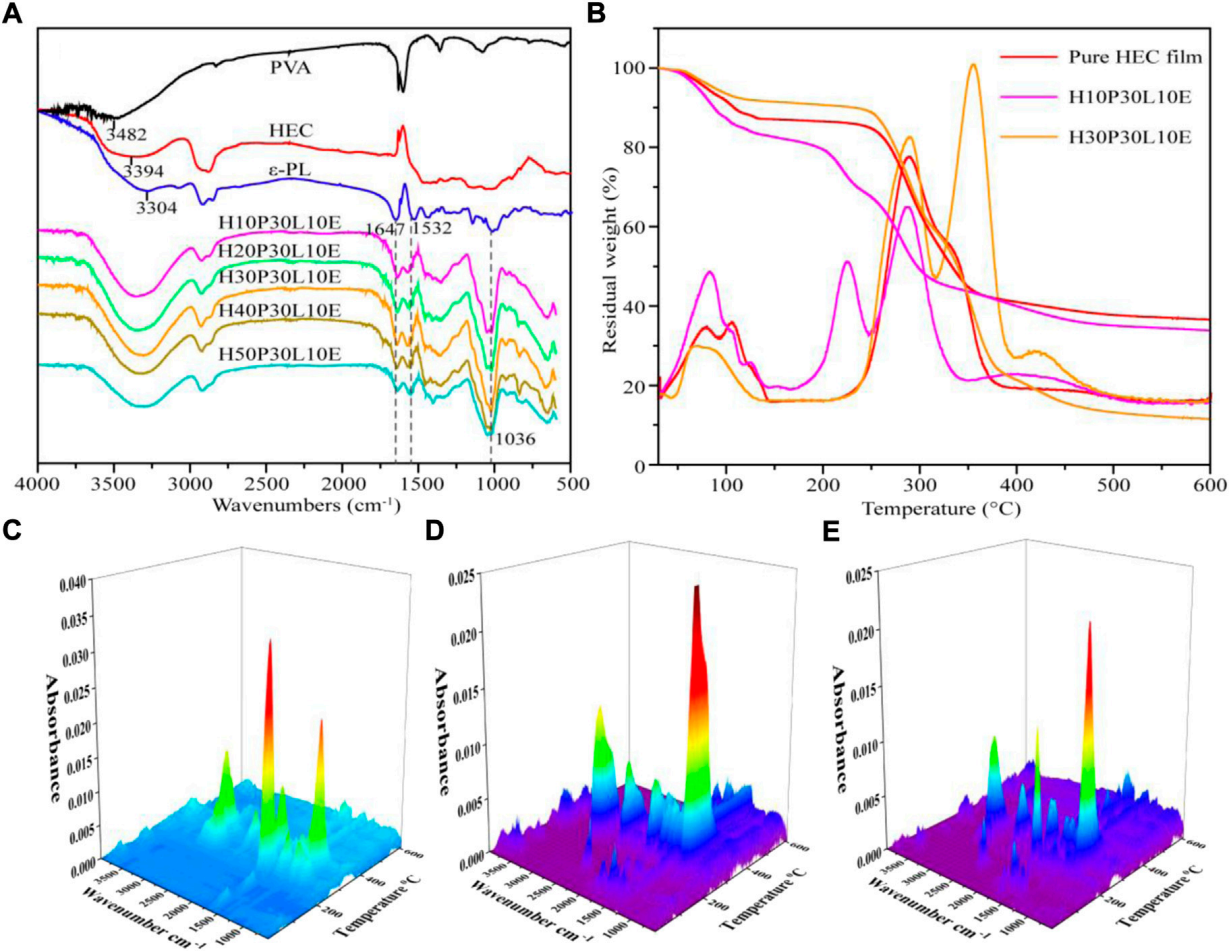
FT-IR spectra **(A)**, TGA-DTG curves **(B)** and three-dimensional surface graphs for the FTIR spectra of the evolved gases produced by pure HEC film **(C)**, H10P30L10E **(D)** and H30P30L10E **(E)** pyrolysis.

Figure 2A shows the FTIR spectra of HEC, PVA, ε-PL, and the composite films reinforced with different PVA contents. For HEC and PVA, the bands at 3394 cm-1 and 3482 cm-1 were assigned to stretching vibrations of -OH, respectively (Zhao et al. 2016). For ε-PL, the bands at 3304, 1647 and 1532 cm-1 were attributed to the N-H/O-H, amide I (C=O) and amide II (N-H), respectively (Gao et al. 2014; Wahid et al. 2019). Additional peaks observed due to the incorporation of PVA and ε-PL in the spectra of the composite films confirmed that the cross-linking of PVA and ε-PL with HEC were successfully synthesized. Obviously, it was observed that the absorption bands of -OH of HEC and PVA shifted to a lower absorption area in the composite films, confirming the formation of cross-linking network between the -OH groups of HEC and PVA. Moreover, by increasing PVA content, this shifting strengthened, while the intensity of the -OH bands decreased. Furthermore, the C-O-C stretching vibration at 1036 cm-1 in FTIR spectra of the composite films increased (Zhao et al. 2016). The above results indicated the intensified cross-linking effect by increasing PVA content.

Thermal stability of pure HEC film and the composite films were studied by TGA/DTG, as shown in Figure 2B. The initial weight loss in the temperature range of 50–150°C is attributed to evaporation of water. Comparatively, H10P30L10E showed the higher moisture content than pure HEC film, indicating its increased hydrophilicity. For H30P30L10E, its moisture content decreased, which could be attributed to the increased hydrogen bonding interactions developed its hydrophobicity. Pure HEC film showed one main degradation peak at 285°C. After reinforcing with 10% PVA, two degradation peaks occurred at 219 and 280°C, corresponding to the removal of the oxygen functional groups from the PVA molecules and the formation of the polyene intermediate and the degradation of HEC main chain (Tanpichai and Oksman 2016), respectively, indicating the decreased thermal stability of H10P30L10E. Increasing PVA content to 30%, these two peaks occurred at 290 and 354°C, indicating the developed thermal stability of H30P30L10E. Additionally, the DTG curves of the composite films showed degradation stage in the range of 400–450°C, which could be assigned to the decomposition of main chain of PVA (Kumar et al., 2019).

The gaseous products of TG were investigated by FTIR in order to better understand the pyrolysis mechanism of the composite films. The three-dimensional FTIR spectrograms of pure HEC film, H10P30L10E and H30P30L10E were listed in Figure 2. According to the Lamber-Beer Law, the concentration of release products was linearly reflected on the intensity of absorbance at specific wavenumber. So the IR peak heights represented the generated concentration of gaseous products (Xu F.-x. et al., 2020). It could be clearly observed that pure HEC film mainly degraded at temperatures of 200–400°C, producing a large proportion of CO_2_ (2359 cm^−1^), C=O (1748 cm^−1^, ketone, aldehydes, carboxylic acid, esters), C-OC/C-C (1037 cm^−1^) and C=C (1513 cm^−1^, alkene, aromatics), with a small amount of H_2_O (3428 cm^−1^) and CH_4_ (2821 cm^−1^) evolving (Xu F.-x. et al., 2020). After reinforcing with PVA, the characteristic absorbance heights corresponding to–CH_3_ (1250 cm^−1^) and–OH (3310 cm^−1^) increased, further indicating the increased hydrogen bonding interactions.

### 3.3 X-ray diffraction

**FIGURE 3:**
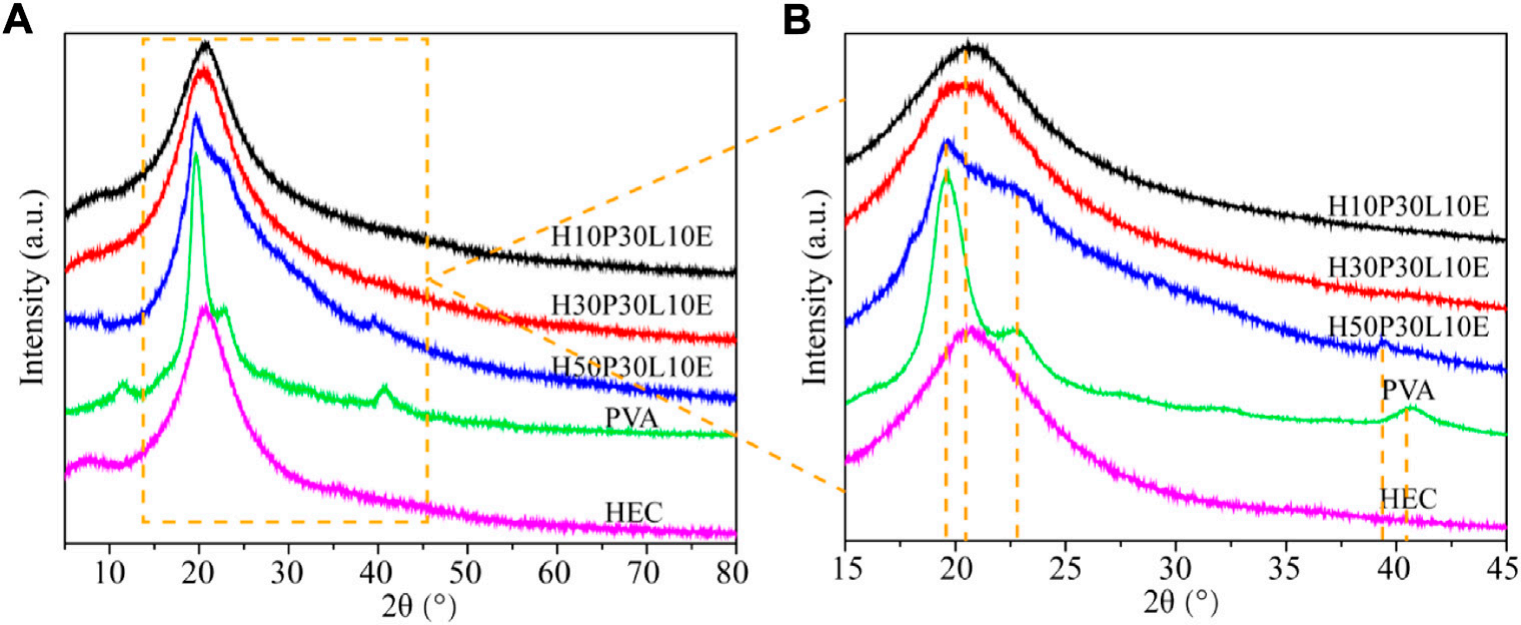
XRD patterns **(A,B)** of HEC, PVA and the composite films reinforced with different PVA contents.

X-Ray diffraction study was conducted to examine the change in the intensity of the characteristic peaks (Jo et al., 2021). As shown in Figure 3, HEC has two specific diffraction peaks at 2θ = 8.04° and 20.55° (Jin et al., 2018). PVA shows characteristic diffraction peaks at 2θ = 19.61°, 22.93° and 40.65°, indicating its semi-crystalline nature (Ali et al., 2021). The XRD patterns of H10P30L10E and H30P30L10E show only one broad diffraction peak at 2θ = 20.74°, whereas the other diffraction peaks of HEC and PVA are disappeared. This result indicated the good compatibility and miscibility between the two materials, which may be attributed to the interactions formed between -OH groups originating from HEC and PVA. Increasing PVA content to 50%, the reflection peak positions of H50P30L10E exhibit some changes. The diffraction peaks of HEC in H50P30L10 were relatively broad with high intensity. In addition, the characteristic peaks of PVA were shifted and were broader than those of pure PVA. This result indicated that PVA could interact with HEC to break their original crystalline structure and form a new crystalline region. Further, the high degree of crystalline of H50P30L10 is responsible for the low swelling ratio of film. Similar results were observed for cellulose pulp/HEC/turmeric powder (Jo et al., 2021), cellulose/PVA (Wahid et al., 2019) and xylan/PVA (Zhang et al., 2020) based composites.

### 3.4 Morphology analyses

**FIGURE 4:**
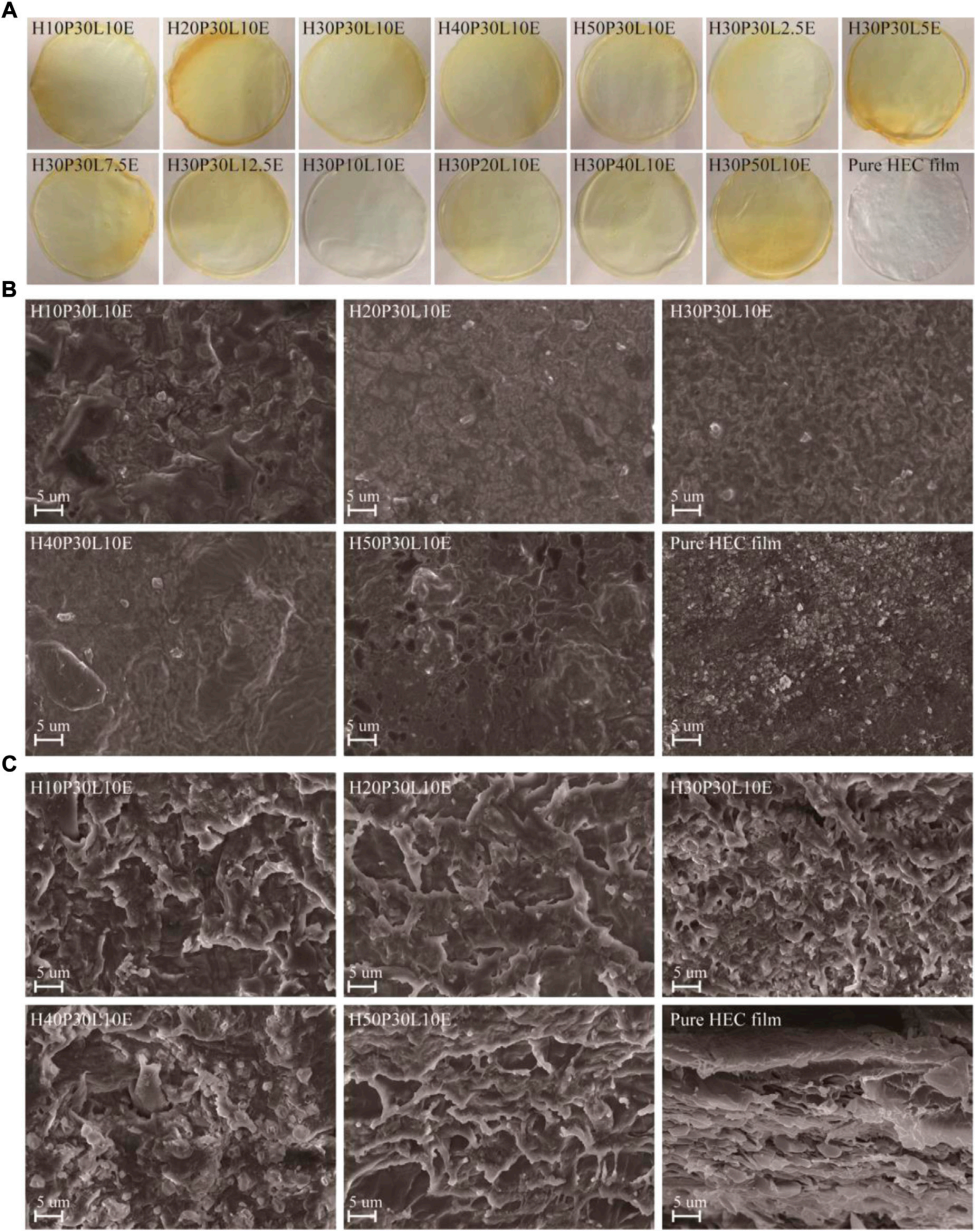
Digital photograph of the composite films **(A)**; Surface **(B)** and cross-section **(C)** morphologies of films obtained from SEM.

Physical appearance is the most intuitive property of the film materials. From Figure 4A, visual analysis indicated that pure HEC film was transparent, while the composite films were yellowish in color, due to the color of ε-PL (Wahid et al., 2019). By varying PVA contents, ECH and ε-PL did not affect the color of the composite films. This could be attributed to the homogenous dispersion of the components in film matrix.

The surface (Figure 4B) and cross cross-sectional (Figure 4C) morphologies of the composite films reinforced with different PVA contents were investigated by SEM analysis. From Figure 4B, pure HEC film had a coarse surface morphology with apparent agglomeration. Comparatively, the prepared composite films showed a relative smooth appearance. Cross-linking with PVA consumed the–OH groups of HEC, that is, decreased the inter- and intra-hydrogen bondings interactions of HEC as well as its interactions with water upon the drying process. From Figure 4C, pure HEC film presented an irregular and sheet-like skeleton morphology. A different morphology was observed when PVA was added to the film matrix. The cross-sectional images of the composite films showed a compact and interconnected morphology, and without any evidence of phase separation, as expected for a homogenous material. This could be attributed to the intermolecular polymer associations by the chemical cross-linking among HEC, PVA, ECH and ε-PL. Moreover, the interconnected morphology of the composite films became apparent by increasing PVA contents. This suggested that PVA offered chemical cross-linking with HEC, in addition to being reinforcement in the polymer matrix (Indumathi et al., 2019).

### 3.5 UV-shielding and transparency of the composite films

**FIGURE 5:**
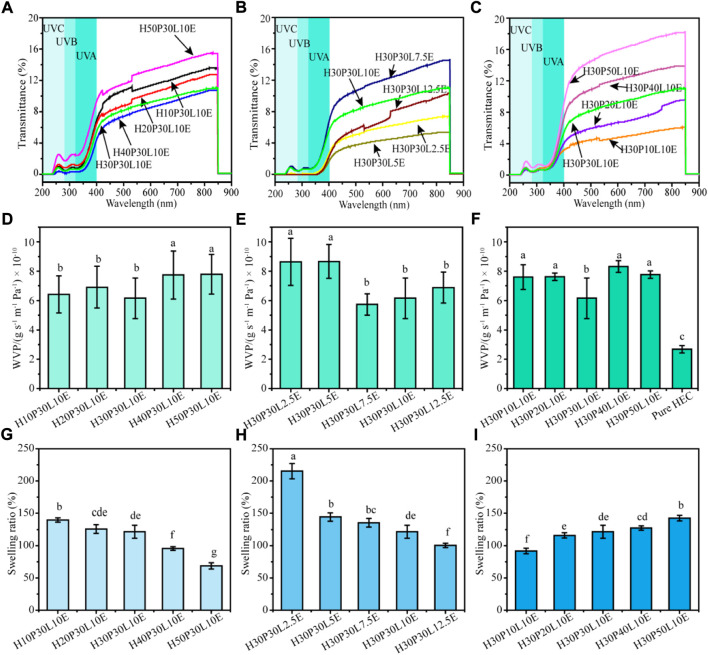
UV‐Vis light transmittance spectra **(A,B,C)**, water vapor permeability **(D,E,F)** and swelling ratio **(G,H,I)** of the composite films.

UV light can generate singled oxygen that degrades antioxidants and vitamins and accelerates the oxidation of lipids (Sirviö et al., 2020). Film with desirable UV-shielding capability plays key role in food packaging because blocking UV radiation helps to prevent the spoilage of food. Therefore, the UV-shielding and transmittance of the composite films were investigated in the wavelength range of 200–900 nm, and the results were presented in Figure 5. The visible light transmittance values of the composite films were below 20% at 600 nm. This could be due that ε-PL was yellowish in color which could reflect more light, or the formed chemical cross-linking in the composite films could disrupt the crystalline structure of HEC, making them more difficult to pass light through it (Wahid et al., 2019). For all the films tested, strong absorption can be observed in the range of 250–350 nm, associated with the presence of ε-PL (Guo et al., 2020). Due to the strong absorption, the transmittance of UV light (<400 nm) was practically below 4% in all the films, indicating that the composite films are good UV-shielding materials. Moreover, Supplementary Table S1 shows the percentage of blocking from UV-A and UV-B and UPF values of the composite films. By varying the contents of PVA, ECH and ε-PL, the UV-shielding ability of the composite films could be well controlled. The optimum values of the percentage of blocking from UV-A and UV-B as well as UPF values were 98.35%, 99.99% and 60.25, respectively.

### 3.6 Water vapor permeability and swelling property

The water barrier ability of films plays an important role in food packaging area. For dry food, the moisture barrier ability is required to protect food from moisture deterioration. For fresh food, film is used to retain the moisture and avoid dehydration (Priyadarshi et al., 2018). Therefore, the WVP was determined to evaluate the effect of the barrier properties on the composite films (Figure 5). The WVP of pure HEC film was 2.86 × 10^–10^ g s^−1^ m^−1^ Pa. After cross-linking with PVA, the WVP of the composite films generally increased. Based on the “adsorption-diffusion-desorption” (Xu J. H. et al., 2020), the incorporation of hydrophilic PVA and ε-PL improved the opportunity of adsorbing water molecules, which is the fundamental cause of the increased WVP values of the composites film. Moreover, the cross-linking reaction may break the crystalline structure of HEC, which will increase the solubility and permeability of water. From Figure 5D, increasing PVA content from 10% to 20% resulted in an increase of WVP. Further increase PVA content to 30%, the WVP value decreased to 6.16 × 10^–10^ g s^−1^ m^−1^ Pa (*p* < 0.05), which was due that the increased chemical cross-linking could block the water vapor micro-paths in the film network. Additionally, the effective dispersion of PVA in HEC matrix helps to facilitate the formation of a torturous path. This result correlates well with the SEM analysis. However, increasing PVA content to 50%, the WVP of film apparently developed, probably due to the strong hydrophilic nature of PVA. From Figures 5E,F, similar variation trends could be observed by changing the contents of ε-PL and ECH.

**FIGURE 6:**
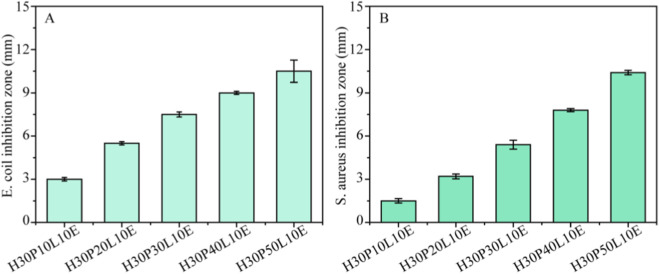
Antibacterial activity of the composite films against *E. coli* and *S. aureus*.

The interaction of the composite films with water was further investigated in terms of swelling behavior. For pure HEC film, it is difficult to calculate its swelling ratio due to its good solubility in water. As shown in Figure 6, the swelling property of the composite films depends on its composition. From Figure 5G, the swelling ratio decreased from 139.80% for H10P30L10E to 68.77% for H50P30L10E. This declination could be attributed to the reinforcing effect of PVA which formed a strong chemical cross-linking with HEC matrix and hindered the water uptake of the composite films (Gan et al., 2021). From Figure 5H, H30P30L2.5E exhibited the highest swelling ratio with 2.5% ECH. Increasing ECH content to 12.5%, the swelling ratio of the composite films dramatically declined. ECH is a typical hydroxyl cross-linking agent, and the above results indicated that ECH could combine the components tightly to reduce the exposed hydroxyl groups and decrease the availability of hydroxyl groups to react with water molecules (Ma et al., 2022); thus, the swelling ratio of the composite films decreased. The swelling property of ECH cross-linked composite films agreed well with Ma et al. (Ma et al., 2022) who reported the development in the water resistance of ECH cross-linked cellulose/glucomannan/lignin composite films. From Figure 5I, however, the swelling ratio of the composite films developed from 91.64% to 142.54% by increasing ε-PL content from 10% to 50%. According to previous studies (Wu et al., 2019; Xu J. H. et al., 2020), the hydrophilicity of ε-PL could result in an increase of the wettability of film. Apart from that, the addition of ε-PL may disrupt the integrity of film matrix during the drying process, which led to a faster diffusion of water into films. Based on the aforementioned results, we speculated the outcomes of interactions among HEC, PVA, ECH and ε-PL in the composite films could determine the film network structure and thus regulate the WVP and swelling ratio.

### 3.7 Antibacterial properties

ε-PL is promising antibacterial agents which has pronounced antibacterial activity against different types of bacteria.

The inhibitory effects of the composite films on the growth of the two kinds of food pathogen, *E. coil* and *S. aureus*, were shown in Figure 6. As expected, the composite films possessed considerable antibacterial activity after addition ε-PL. Moreover, the antibacterial activity of the composite films increased by increasing ε-PL content. It has been reported that the positively charged amino groups in ε-PL could interact with negatively charged microbial cell membranes through electrostatic interaction, and eventually resulted in bacterial death. Comparatively, the inhibition efficiency of *E. coli* was higher than that of *S. aureus*, probably due to different cell surface conditions of the tested bacterial membrane (Wu et al., 2019).

### 3.8 *In vitro* cytotoxicity

**FIGURE 7:**
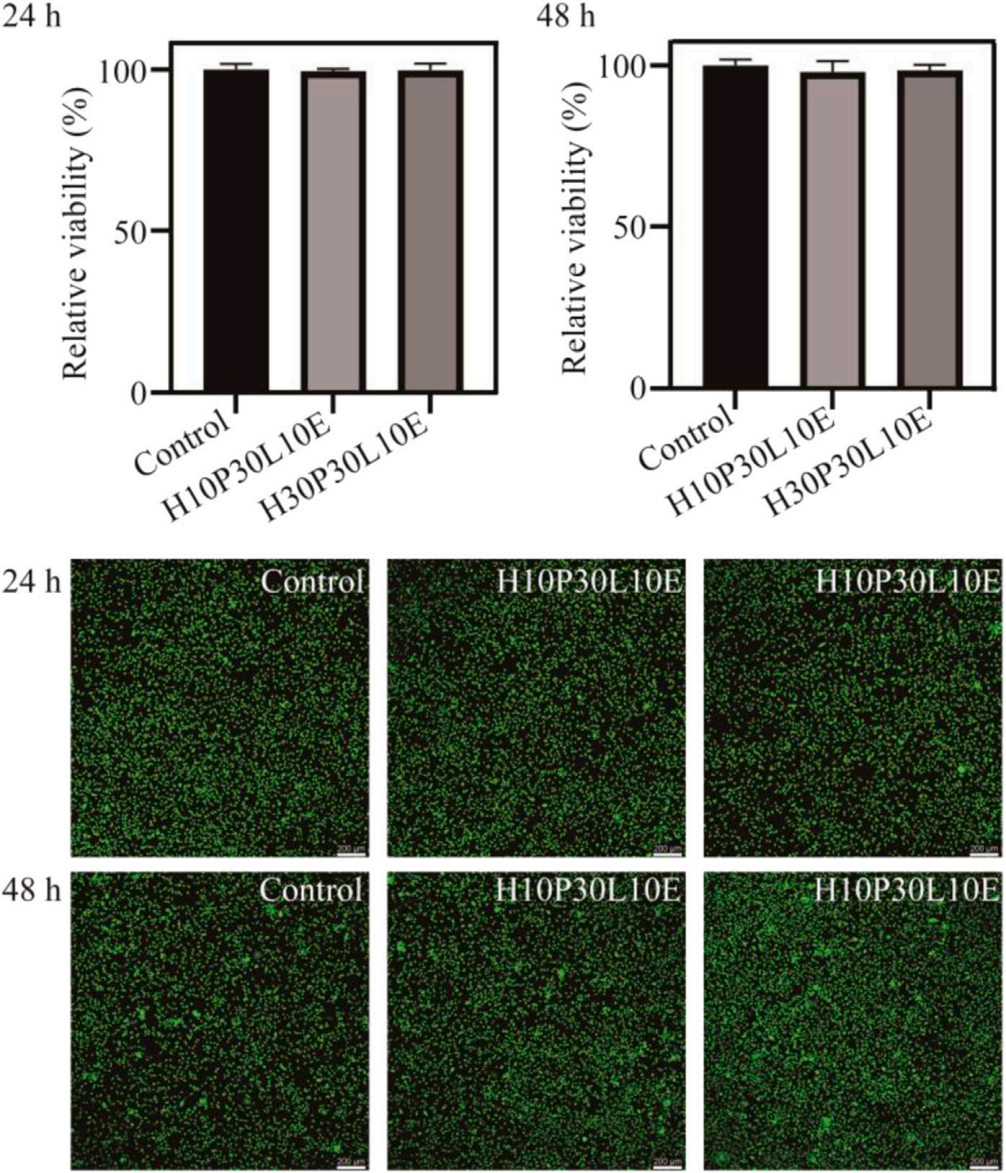
Cell viability results of the NIH‐3T3 cells of H10P30L10E and H30P30L10E composite films after 24 and 48 h.

Biocompatibility is an important property for food packaging materials. *In vitro* cytotoxicity studies were performed to investigate the effect of the composite films on proliferation of NIH-3T3 cells. As shown in Figure 7, none of the tested composite films showed a significant effect on the proliferation of NIH-3T3 cells and displayed the cell growth above 98% compared to the untreated control (100%). Further, no significant difference in cell morphology was observed in the untreated control, H10P30L10E and H30P30L10E composite films. These observations indicated the prepared composite films are safe for use in food packaging area.

### 3.9 Packaging experiment of grape

**FIGURE 8:**
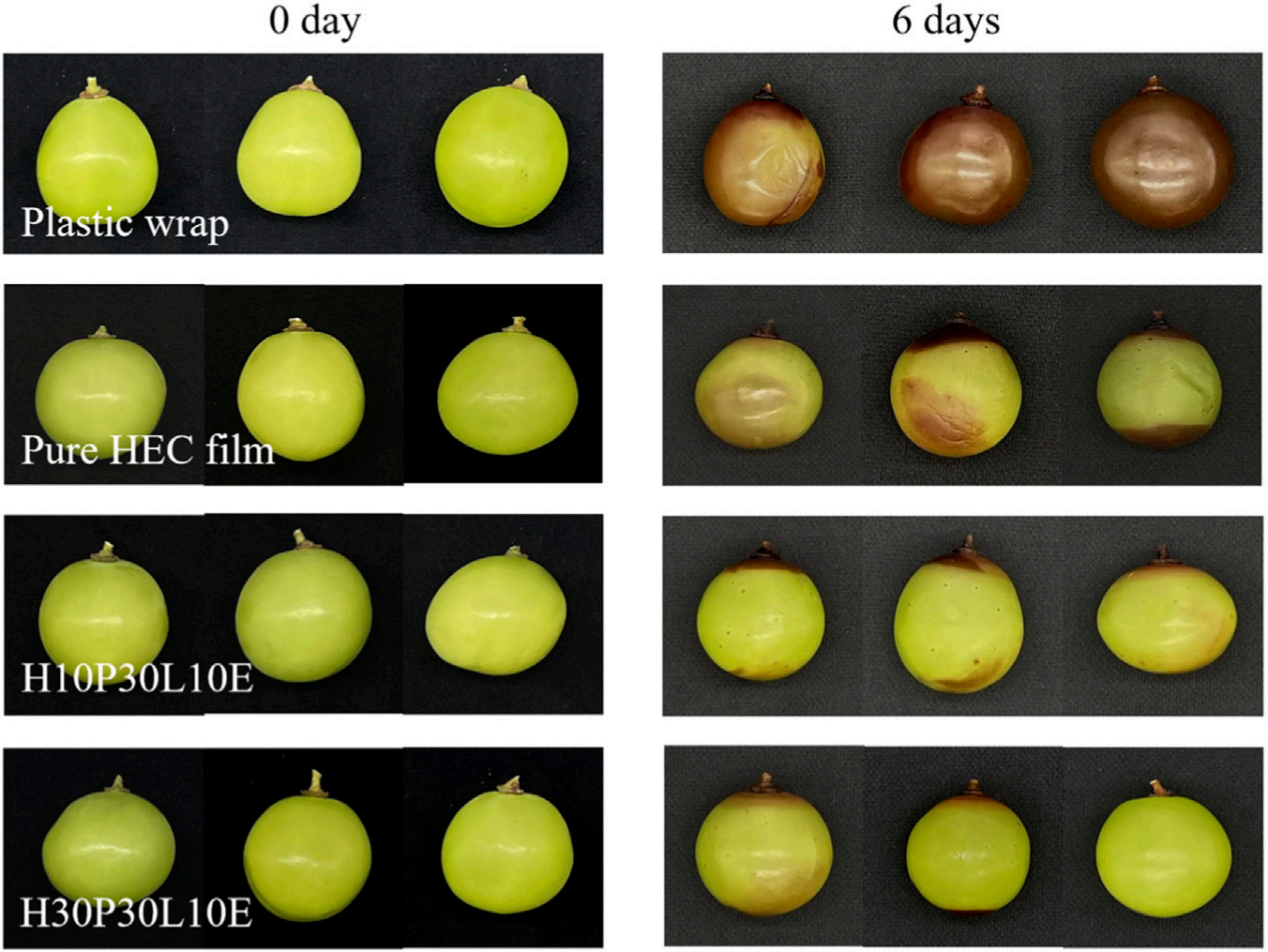
Photograph of fresh grape and preserved grapes after 6 days.

The appearance of the fresh and preserved grapes are shown in Figure 8, and the changes of the weight loss, hardness, total soluble solids (TSS) and color of grapes are listed in Supplementary Table S2. Plastic wrap stored grapes became shriveled and dehydrated after 6 days due to the susceptibility of grape to water loss during storage. And the color of grapes became brownish black. At this point, all grapes stored using plastic wrap were below marketable quality. In comparison, pure HEC film stored grapes showed slight deterioration of texture in terms of color and moisture content. After reinforcing with PVA, no apparent shrinkage evidenced by the little moisture loss was observed for H10P30L10E and H30P30L10E. And the color of grapes still remained fresh green during the whole preservation time period. This behavior was more distinct for H30P30L10E. This was probably due to the good barrier property and antibacterial property of the prepared composite films, broadening its application in food packaging area.

## 4 Conclusion

ECH-cross-linked HEC composite films were prepared using PVA and ε-PL as functional components. The structure and morphology of the composite films were characterized by TG-FTIR, XRD and SEM. The results indicated that chemical cross-linking with PVA and ε-PL significantly influenced the mechanical properties and the morphology structure of the composite films. The maximum tensile strength and elongation at break of the composite film reached to 95.9 ± 4.1 MPa and 148.8 ± 2.6%, respectively. The composite films were semi-transparent and yellowish in color, and had good UV-shielding capability with both UV-A and UV-B blocking percentage over 98%. The chemical cross-linking formed among the components endowed films with moderate water vapor permeability and swelling ability. Additionally, the composite films exhibited good antibacterial ability and biocompatibility. In terms of these good comprehensive properties of the prepared HEC-based composite films, the shelf life of grapes were extended, indicating its promising applications in food packaging area.

## Data Availability

The original contributions presented in the study are included in the article/Supplementary Material, further inquiries can be directed to the corresponding authors.
