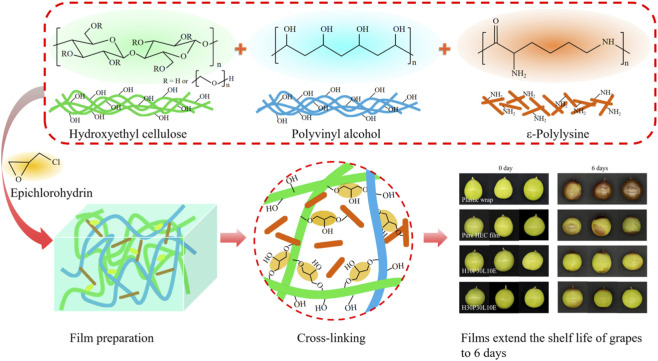# Correction: Development of functional hydroxyethyl cellulose-based composite films for food packaging applications

**DOI:** 10.3389/fbioe.2026.1839638

**Published:** 2026-05-08

**Authors:** Xueqin Zhang, Haoqi Guo, Wenhan Luo, Guojian Chen, Naiyu Xiao, Gengsheng Xiao, Chuanfu Liu

**Affiliations:** 1 College of Light Industry and Food Technology, Zhongkai University of Agriculture and Engineering, Guangzhou, China; 2 Academy of Contemporary Agricultural Engineering Innovations, Zhongkai University of Agriculture and Engineering, Guangzhou, China; 3 Guangdong Key Laboratory of Science and Technology of Lingnan Specialty Food, Zhongkai University of Agriculture and Engineering, Guangzhou, China; 4 State Key Laboratory of Pulp and Paper Engineering, South China University of Technology, Guangzhou, China

**Keywords:** hydroxyethyl cellulose, chemical cross-linking, composite film, UV-shielding, antibacterial activity

In the published article, there was an error in Figure 6 as published. The *Escherichia coli* antibacterial pictures of the H30P20L10E and H30P40L10E in Figure 6 were incorrect. The corrected Figure 6 and its caption appear below.

**FIGURE 6:**
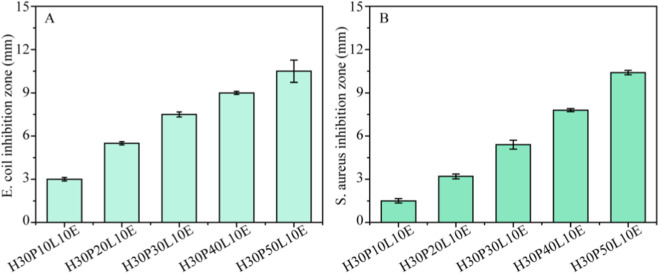
Antibacterial activity of the composite films against *E. coli* and *S. aureus*.

In the published article, there was an error in Figure 8 as published. The grapes’ preserving pictures of the pure HEC film at day 0 in Figure 8 were incorrect. The corrected Figure 8 and its caption appear below.

**FIGURE 8:**
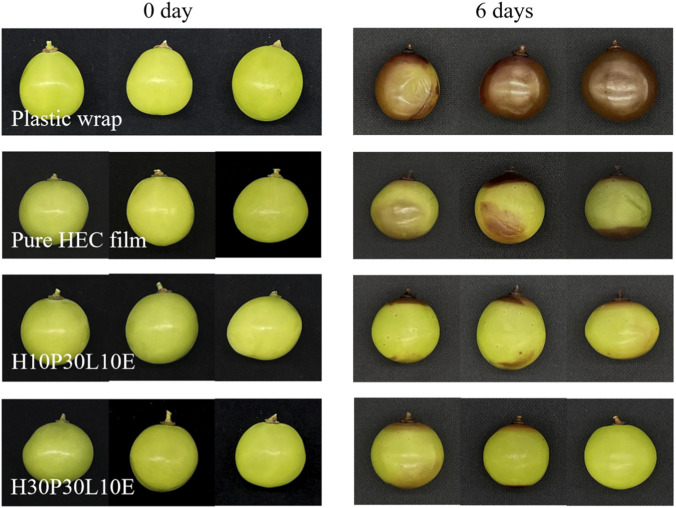
Photographs of fresh grapes and preserved grapes after 6 days.

In the published article, there was an error in the Graphical Abstract as published. The grapes’ preserving pictures of the pure HEC film at day 0 in the Graphical Abstract were incorrect. The corrected Graphical Abstract and its caption appear below.

The original article has been updated.

**GRAPHICAL ABSTRACT:**