# Supplementary material for: Development of functional hydroxyethyl cellulose-based composite films for food packaging applications
Source: Front Bioeng Biotechnol. 2022 Sep 29;10:989893. doi: 10.3389/fbioe.2022.989893 (PMC9557200; doi:10.3389/fbioe.2022.989893)
Supplement: Supplementary file 1 [file DataSheet1.docx]

Supplementary Material

Development of Functional Hydroxyethyl Cellulose-based Composite Films for Food Packaging Applications

**Xueqin Zhang^1,2,3,4^, Haoqi Guo^1^, Wenhan Luo^1,2,3^, Guojian Chen^1,2,3^, Naiyu Xiao^1,2,3,*^, Gengsheng Xiao^1,2,3,*^, Chuanfu Liu^4,*^**

^1^College of Light Industry and Food Technology, Zhongkai University of Agriculture and Engineering, Guangzhou 510225, China

^2^Academy of Contemporary Agricultural Engineering Innovations, Zhongkai University of Agriculture and Engineering, Guangzhou 510225, China

^3^Guangdong Key Laboratory of Science and Technology of Lingnan Specialty Food, Zhongkai University of Agriculture and Engineering, Guangzhou 510225, China

^4^State Key Laboratory of Pulp and Paper Engineering, South China University of Technology, Guangzhou 510640, China

*** Correspondence:**Naiyu Xiao
[xiaony81@163.com](mailto:xiaony81@163.com)

Gengsheng Xiao
Gshxiao@aliyun.com

Chuanfu Liu
chfliu@scut.edu.cn

# Supplementary Tables

**Table S1** The contents of PVA, ECH and ε-PL in the composite films, and the percentage of blocking from UV-A and UV-B, and UPF values of the composite films.

| Film | PVA  (%) | ECH  (%) | ε-PL  (%) | T_600_ (%) | Percentage blocking | | UPF value |
| --- | --- | --- | --- | --- | --- | --- | --- |
|  |  |  |  |  | UV-A | UV-B |  |
| H10P30L10E | 10 | 10 | 30 | 11.38 | 93.80 | 99.66 | 15.29 |
| H20P30L10E | 20 | 10 | 30 | 10.30 | 94.56 | 99.74 | 17.54 |
| H30P30L10E | 30 | 10 | 30 | 15.92 | 93.37 | 99.24 | 13.53 |
| H40P30L10E | 40 | 10 | 30 | 8.54 | 95.94 | 99.82 | 23.58 |
| H50P30L10E | 50 | 10 | 30 | 13.31 | 93.31 | 99.27 | 13.48 |
| H30P30L2.5E | 30 | 2.5 | 30 | 5.98 | 97.38 | 99.99 | 38.02 |
| H30P30L5E | 30 | 5 | 30 | 4.31 | 98.35 | 99.99 | 60.25 |
| H30P30L7.5E | 30 | 7.5 | 30 | 12.01 | 94.21 | 99.78 | 16.64 |
| H30P30L12.5E | 30 | 12.5 | 30 | 6.64 | 97.58 | 99.99 | 41.52 |
| H30P10L10E | 30 | 10 | 10 | 4.74 | 97.65 | 99.48 | 34.60 |
| H30P20L10E | 30 | 10 | 20 | 6.63 | 97.65 | 99.68 | 26.32 |
| H30P40L10E | 30 | 10 | 40 | 12.06 | 93.11 | 99.75 | 14.01 |
| H30P50L10E | 30 | 10 | 50 | 15.92 | 92.49 | 99.73 | 12.85 |

**Table S2** Effect of different films treatments on the storage quality of grapes stored at 25 °C and 55% RH for 6 days.

| Film | Weight loss (%) | Hardness (N) | TSS (%) | L* | a* | b* |
| --- | --- | --- | --- | --- | --- | --- |
| PE | 7.02 ± 0.34 | 0.82 ± 0.36 | 11.47 ± 1.95 | 15.50 ± 2.97 | 3.51 ± 2.55 | 21.83 ± 5.96 |
| Pure HEC film | 5.48 ± 1.78 | 1.96 ± 0.35 | 17.37 ± 0.98 | 30.32 ± 0.87 | -8.04 ± 0.68 | 27.64 ± 1.92 |
| H10P30L10E | 5.84 ± 3.15 | 1.82 ± 0.39 | 17.13 ± 0.42 | 26.25 ± 2.14 | -5.57 ± 3.71 | 17.88 ± 2.53 |
| H30P30L10E | 6.61 ± 0.76 | 2.05 ± 0.48 | 18.87 ± 1.37 | 28.93± 0.67 | -7.36 ± 0.50^c^ | 25.30 ± 3.47 |
